# A machine learning-based diagnostic model for children with autism spectrum disorders complicated with intellectual disability

**DOI:** 10.3389/fpsyt.2022.993077

**Published:** 2022-09-21

**Authors:** Chao Song, Zhong-Quan Jiang, Li-Fei Hu, Wen-Hao Li, Xiao-Lin Liu, Yan-Yan Wang, Wen-Yuan Jin, Zhi-Wei Zhu

**Affiliations:** ^1^Department of Developmental and Behavioral Pediatrics, The Children’s Hospital, Zhejiang University School of Medicine, National Clinical Research Centre for Child Health, Hangzhou, China; ^2^School of Public Health, Lanzhou University, Lanzhou, China

**Keywords:** artificial intelligence, machine learning, child, autism spectrum disorder, intellectual disability, diagnostic model

## Abstract

**Background:**

Early detection of children with autism spectrum disorder (ASD) and comorbid intellectual disability (ID) can help in individualized intervention. Appropriate assessment and diagnostic tools are lacking in primary care. This study aims to explore the applicability of machine learning (ML) methods in diagnosing ASD comorbid ID compared with traditional regression models.

**Method:**

From January 2017 to December 2021, 241 children with ASD, with an average age of 6.41 ± 1.96, diagnosed in the Developmental Behavior Department of the Children’s Hospital Affiliated with the Medical College of Zhejiang University were included in the analysis. This study trained the traditional diagnostic models of Logistic regression (LR), Support Vector Machine (SVM), and two ensemble learning algorithms [Random Forest (RF) and XGBoost]. Socio-demographic and behavioral observation data were used to distinguish whether autistic children had combined ID. The hyperparameters adjustment uses grid search and 10-fold validation. The Boruta method is used to select variables. The model’s performance was evaluated using discrimination, calibration, and decision curve analysis (DCA).

**Result:**

Among 241 autistic children, 98 (40.66%) were ASD comorbid ID. The four diagnostic models can better distinguish whether autistic children are complicated with ID, and the accuracy of SVM is the highest (0.836); SVM and XGBoost have better accuracy (0.800, 0.838); LR has the best sensitivity (0.939), followed by SVM (0.952). Regarding specificity, SVM, RF, and XGBoost performed significantly higher than LR (0.355). The AUC of ML (SVM, 0.835 [95% CI: 0.747–0.944]; RF, 0.829 [95% CI: 0.738–0.920]; XGBoost, 0.845 [95% CI: 0.734–0.937]) is not different from traditional LR (0.858 [95% CI: 0.770–0.944]). Only SVM observed a good calibration degree. Regarding DCA, LR, and SVM have higher benefits in a wider threshold range.

**Conclusion:**

Compared to the traditional regression model, ML model based on socio-demographic and behavioral observation data, especially SVM, has a better ability to distinguish whether autistic children are combined with ID.

## Introduction

Autism spectrum disorder (ASD) is a neurodevelopmental disorder characterized by social disorder and restricted, repetitive, stereotyped behavior (1). The worldwide prevalence of ASD has increased year by year. According to the monitoring data released by the United States in 2021, the prevalence of ASD is higher than 2.27%, that is, one in 44 children has ASD. ASD has become one of the fastest-growing diseases in children and a public health problem threatening children’s health (2–4), which not only affects the life quality of children but also increases the economic burden on society and families due to the high cost of the intervention (5).

The proportion of ASD combined with intellectual disability (ID) is about 33–35% (2, 6), with more comorbidities, such as epilepsy and self-injurious behavior (7, 8), and more medical expenses (9), but they may be less effective in social skills training through behavioral interventions (10). Thus, the basic intelligence quotient (IQ) level of children with ASD affects the intervention effect (11). Since the dose-response relationship between weekly intervention duration (dose) and IQ scores (response) was confirmed (12), increasing the intensity of the intervention for ASD combined with ID can be considered. It is also critical to identify children with autism of average intelligence, as their adaptive functioning lags behind their IQ (13–15). Early identification and intervention for children with autism of average intelligence can improve social and vocational outcomes in this population (16). In terms of intervention content, training in social adjustment should be as important as social intervention for children with autism who have an IQ greater than 70, while training in cognitive skills is also important for children with autism with comorbid ID. Not only that, but the intelligence level of children with ASD is also related to their emotions. Anxiety is the most common emotional problem in children with ASD, but in most cases it is difficult to distinguish the symptoms of anxiety from those of ASD (17). Although anxiety and depression also frequently occur in children with ASD who have normal IQ (17, 18), identifying emotional problems such as anxiety in children with ASD combined with ID can be more difficult (19). Therefore, intellectual assessment of children with ASD may help in the early detection of their emotion-related problems. Our clinical experience also suggests that the goal of intervention is to promote integration into mainstream society for most children with normal abilities, while working toward self-care for most children with low abilities. Overall, its judgment of an autistic child’s IQ informs the planning of individualized interventions.

Wechsler Intelligence Scale for Children-IV (WISC-IV) is the most commonly used tool for evaluating intelligence levels (20), and it is also considered to be suitable for children on the ASD (21). However, the use and scoring of WISC-IV need to be authorized by relevant parties, with high acquisition costs, many standardized test items, and specific training for evaluators, which hinder the primary medical workers from evaluating the intelligence level of children with ASD. Furthermore, medical and health resources distribution is uneven in developing countries like China (22), with millions of children with ASD (23). Therefore, it is unrealistic to carry out comprehensive intelligence assessments like WISC-IV. Therefore, it is particularly urgent to develop a simple and effective diagnostic model for primary pediatric medical workers to identify ID in autistic children. Using behavioral observation results to evaluate the intelligence of children with ASD can simplify the diagnostic process of autistic children with ID (24), and help promote the application at the grass-roots level.

In the research of disease diagnosis, regression analysis is a commonly used diagnostic method (25–29), which is simple and interpretable, such as Cox regression and Logistics regression (LR). However, traditional regression methods mainly deal with low-level relations, making it difficult to analyze high-level non-linear relations (28, 30–32). The correlation between influencing factors and outcomes is often non-linear in epidemiological data. Concurrently, linear regression models are used to fit the non-linear relation, and the results are often biased. Machine learning (ML) is a set of computational methods that can discover complex non-linear relations between inputs and outputs, which has been widely used in disease diagnosis and health research (27, 30, 33, 34). Support Vector Machine (SVM) is a class of ML learners that performs exceptionally well on small sample datasets (35). Ensemble learning is a widely used method with excellent performance (36). The Random Forest (RF) of Bagging ensemble idea and XGBoost of Boosting ensemble idea are two of the most representative models.

The existing predictive diagnosis of ASD or ASD comorbidities (e.g., attention deficit hyperactivity disorder) usually includes four aspects, disease prevention or risk factor identification, disease diagnosis, disease efficacy prediction, and disease prognosis prediction. Most of these diagnostic models use complex diagnostic-related data, such as expensive head MRI, EEG (37–39), and blood biochemical indicators. These data were used to build diagnostic models to diagnose ASD-related disorders and to determine their type or severity. Currently, much of the past research on diagnostic models has focused on diagnostic imaging, neglecting the importance of demographic and behavioral observational data (40). Meanwhile, fewer studies have focused on early diagnosis and screening of ASD combined with ID (41, 42). Furthermore, in the context of healthcare resource shortages and COVID-19 pandemic, the application of simple and effective diagnostic tools geared toward most primary care physicians can greatly reduce the burden on the healthcare system. Thus, early diagnosis can identify at-risk populations and initiate personalized interventions while seeking further help from higher levels of care. This is the most cost-effective approach. Fortunately, with the increased availability of data from cross-sectional pediatric studies in China, both demographic and behavioral observations are available. Using these data, diagnostic models can be constructed to help identify autistic children combined with ID at the early diagnostic stage.

This article intends to combine the behavioral observations and socio-demographic data of children with ASD, apply ML methods to the diagnosis of ID in autistic children, and optimize the diagnostic model through feature selection. Simultaneously, SVM, RF, and XGBoost models are compared with the traditional LR model. Finally, comprehensive use of discrimination, calibration, and decision curve analysis (DCA) to evaluate and screen the optimal diagnostic model provides a new perspective for early diagnosis of autistic children with ID.

## Materials and methods

### Study design and population

This study retrospectively collected ASD data from January 2017 to December 2021 in the department of Developmental and Behavioral Pediatrics, the Children’s Hospital, Zhejiang University School of Medicine. These children with ASD met the diagnostic criteria for ASD in the Diagnostic and Statistical Manual of Mental Disorders, 5th Edition (DSM-5). They were also assessed by the WISC-IV and the Adaptive Behavior Assessment System-II (ABAS-II) for adaptive behavior. Those children who scored below 70 on the WISC-IV and had an adaptive disorder were diagnosed with ASD combined with ID. Socio-demographic information and behavioral observation data were used as variables in this study, and the findings were the result of the WISC-IV assessment (see the Appendix). Socio-demographic information includes gender, age at the time of behavioral observation, parent’s education. The behavioral observation items were formulated concerning Autism Diagnostic Observation Schedule (ADOS). First, the language ability was assessed and divided into three types: pre-verbal and single words, phrase speech, as well as fluent speech. Eleven aspects of behavioral observations include whether ASD children have stereotyped use of words or phrases, pointing/gestures, unusual eye contact, facial expressions toward others, the quality of actively expressing social intentions, unusual sensory interest in-game materials or people, complex mannerisms, unusual, repeated interest or stereotyped behavior, overactivity, negative behaviors, and anxiety (Table 1). The scoring criteria are detailed in the table below. The intelligence level of children with ASD was assessed using WISC-IV, the fourth edition, suitable for children and adolescents aged 6–16 years (20). This study was approved by the Ethics Committee of the Children’s Hospital, Zhejiang University School of Medicine (No. 2022-IRB-014).

**TABLE 1:** Behavioral observation scoring criteria.

Behavioral observation variables	0 point	1 point	2 points
Stereotyped speech	Scarcely	Partial	Many
Pointing/gestures	Many	Partial	Scarcely
Unusual eye contact	Scarcely	Partial	Many
Facial expression	Many	Partial	Scarcely
Social quality	Good	Average	Bad
Unusual sensory interest	Scarcely	Partial	Many
Complex mannerisms	Scarcely	Partial	Many
Repetitive stereotyped behaviors	Scarcely	Partial	Many
Overactivity	Scarcely	Partial	Many
Negative behaviors	Scarcely	Partial	Many
Anxiety	Scarcely	Partial	Many

### Feature selection with Boruta

The choice of variables has a decisive impact on the final model performance. Therefore, this study used the Boruta method to process the variable list, a feature selection method established using a random forest classifier (43). It determined the importance of variables by comparing the correlation between real and shaded features. Traditional feature selection algorithms often use filtering, so it is easy to discard some relevant features to minimize errors. However, Boruta is a wrapper method that can find all feature sets through a fully correlated strategy, so weakly correlated predictors can also be preserved (43).

To quantitatively evaluate the impact of feature selection on the model, we separately constructed models based on all variables and the variables of feature selection and evaluated the contribution of feature selection to the diagnostic model using integrated discrimination improvement index (IDI) (44). Performance improved when IDI was greater than 0. Performance was reduced when IDI was less than 0. When IDI was set to 0, the performance remained constant. Among them, hypothesis testing was used to identify whether changes in model performance were statistically significant (two-sided *P* < 0.05 was considered statistically significant).

### Diagnostic models

#### Logistics regression

This study compares traditional LR to other ML methods using it as a reference model. The binary logistic regression used in the study had only two possible outcome values (low versus high function). The log-odds of values marked “1” in LR are linear combinations of predictors, and LR can be expressed as:


$$p\left(y=1|x\right)=\frac{\exp\left(w^{T}\,x+b\right)}{1+\exp\left(w^{T}\,x+b\right)}.$$

where *x* is the predicted value, *y* is the result, *w* is the weight of each predicted value, and *b* is the intercept.

### Support vector machine

The final decision function of SVM is only determined by a very small number of support vectors (35). Thus, it has good performance on small samples. However, most research problems in the real world are often non-linear. To solve this problem, SVM maps non-linear data to high latitudes through a kernel function, making it linearly separable at high latitudes and having a stable performance. Unfortunately, linear kernels are difficult to analyze in non-linear data, and radial basis function kernels are the most commonly used. Therefore, this study adopts the radial basis function kernel for modeling.

#### Ensemble learning method

This study selects RF and XGBoost as representative ensemble learning models. RF is one of the commonly used Bagging methods. Breiman proposed it in 2001 (32). It can parallelly generate multiple decision tree classifiers and integrate them through voting to realize data classification. The random characteristics are mainly reflected in (1) using the bootstrap method to generate multiple datasets of the same size. (2) The variables in each decision tree node are randomly combined from the list. XGBoost, known as the boost policy algorithm, is another ensemble learning method. The main difference between Boosting and Bagging is that constructing the following weak classifier depends on its previous classifier. XGBoost was proposed by Chen et al. (45), aiming to solve the computational problem when gradient boosting decision tree (GBDT) encounters large data sets. Compared to GBDT, XGBoost improvement uses both the first and second derivatives, which significantly speeds up the computation. Furthermore, XGBoost uses multiple weak classifiers to fit the prediction residuals and integrates all the weak learners to obtain a robust ML model.

### Model derivation and internal validation

The dataset is divided into the training set and test set with a ratio of 7:3, which are used for modeling and model performance evaluation. In the modeling phase, Boruta’s method filters variables, and 10-fold cross-validation and grid search were used to tune hyperparameters. The subjects’ accuracy, precision, sensitivity, specificity, and area under curve (AUC) were used to evaluate the model’s discrimination. The Bootstrap method was used to calculate the 95% confidence interval (95% CI) of AUC, and De-long test was used to compare AUC between diagnostic models. Additionally, each diagnostic model is evaluated for its agreement with the ground truth using a calibration plot. Finally, we performed DCA to provide a reference for selecting the best diagnostic model and clinical practice. DCA is a simple method for evaluating diagnostic models that consider both accuracy and clinical utility, as Andrew et al. proposed in 2006 (46).

Furthermore, we use the SHapley Additive exPlanations (SHAP) method to evaluate the interpretability of the optimal model. The SHAP method is derived from game theory based on the SHAP value, which shows the importance of variables and determines the direction of effects (47, 48).

### Statistical analysis

Continuous variables were expressed as mean ± SD (normal distribution) and median of the interquartile range (IQR, skewed distribution), and categorical variables were expressed as percentages. The χ^2^ test or Fisher’s exact test was used to compare the rates, the *t*-test was used to compare the means of normal distribution, the rank-sum test was used to compare the means of non-normal distribution, and the univariate analysis was used to compare the multi-category distribution. Two-sided *P* < 0.05 was considered statistically significant. All methods in this study were implemented with R 3.6.0 with a random seed set to 123. The Boruta feature selection method is derived from Boruta version 7.0.0. pROC version 1.17.0.1 is used for pairwise comparison of AUC, ROC calculation, and drawing. PredictABEL version 1.2-2 is used for the implementation of the IDI evaluation method; e1071 version is used for model parameter tuning 1.7-6; randomForest version 4.6-14 is used to build random forest; e1071 version 1.7-6 is used to build SVM; XGBoost version 1.2.0.1 is used to build XGBoost.

## Results

### Patient characteristics

A total of 241 children with ASD were included, including 98 with ID, with an average age of 6.41 ± 1.96 (Table 2).

**TABLE 2:** The socio-demographic information and behavioral observations of autistic children.

Variable	Total	Whether or not with ID		*P*-value
		Yes	No	
	241	98	143	–
**Gender**				
Male	202 (83.82%)	75 (76.53%)	127 (88.81%)	0.013
Female	39 (16.18%)	23 (23.47%)	16 (11.19%)	
Age	6.41 ± 1.96	6.01 ± 1.75	6.67 ± 2.05	0.010
**Mother’s education attainment**				
Primary school	12 (4.98%)	6 (6.12%)	6 (4.20%)	0.032
Secondary school	37 (15.35%)	17 (17.35%)	20 (13.99%)	
High school	30 (12.44%)	19 (19.39%)	11 (7.69%)	
College/university	147 (61.00%)	52 (53.06%)	95 (66.43%)	
Graduate and above	15 (6.22%)	4 (4.08%)	11 (7.69%)	
**Father’s education attainment**				
Primary school	6 (2.49%)	2 (2.04%)	4 (2.80%)	0.971
Secondary school	53 (21.99%)	24 (24.49%)	29 (20.28%)	
High school	36 (14.94%)	12 (12.24%)	24 (16.78%)	
College/university	133 (55.19%)	50 (51.02%)	73 (51.05%)	
Graduate and above	23 (9.54%)	10 (10.20%)	13 (9.09%)	
**Language ability**				
Pre-verbal/single words	42 (17.43%)	28 (28.57%)	14 (9.79%)	<0.001
Phrase speech	122 (50.62%)	65 (66.33%)	57 (39.86%)	
Fluent speech	77 (31.95%)	5 (5.10%)	72 (50.35%)	
**Behavioral observation**				
Stereotyped speech	1.00 (0.00, 1.00)	1.00 (0.00, 1.25)	1.00 (0.00, 1.00)	0.002
Pointing/gestures	2.00 (1.00, 2.00)	2.00 (2.00, 2.00)	2.00 (1.00, 2.00)	<0.001
Unusual eye contact	2.00 (0.00, 2.00)	2.00 (2.00, 2.00)	2.00 (0.00, 2.00)	0.002
Facial expression	1.00 (0.00, 1.00)	1.00 (0.00, 1.00)	1.00 (0.00, 1.00)	0.002
Social quality	1.00 (1.00, 2.00)	2.00 (1.00, 2.00)	1.00 (1.00, 1.00)	<0.001
Unusual sensory interest	0.00 (0.00, 1.00)	0.00 (0.00, 1.25)	1.00 (0.00, 1.00)	0.001
Complex mannerisms	0.00 (0.00, 0.00)	0.00 (0.00, 0.00)	0.00 (0.00, 0.00)	0.026
Repetitive stereotyped behaviors	1.00 (0.00, 2.00)	1.00 (0.00, 1.00)	2.00 (1.00, 2.00)	<0.001
Overactivity	0.00 (0.00, 0.00)	0.00 (0.00, 0.00)	0.00 (0.00, 0.00)	0.059
Negative behaviors	0.00 (0.00, 0.00)	0.00 (0.00, 0.00)	0.00 (0.00, 0.00)	0.319
Anxiety	0.00 (0.00, 0.00)	0.00 (0.00, 0.00)	0.00 (0.00, 0.00)	0.721

### Selection of predictors using Boruta

**FIGURE 1:**
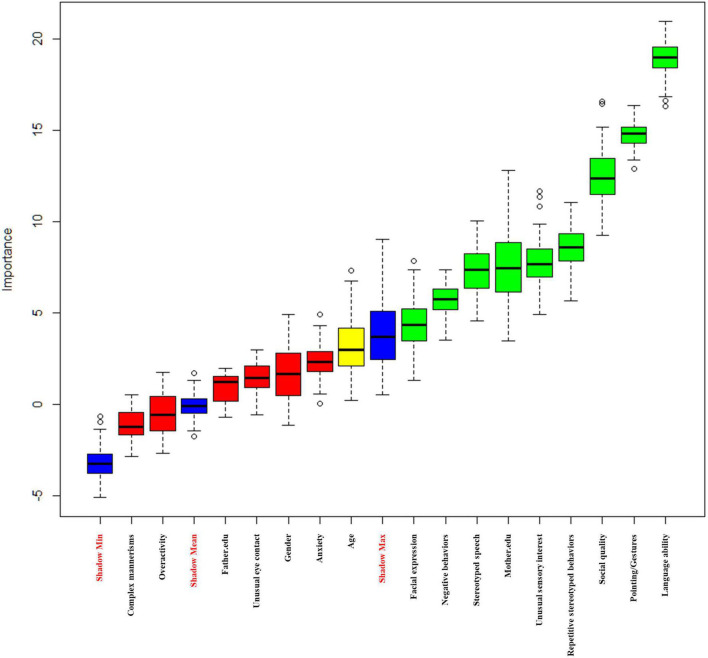
Boruta-based feature selection results.

The Boruta-based feature selection results are displayed in Figure 1. The three blue features represent the maximum *Z*-score, average *Z*-score, and minimum *Z*-score of the shadow feature. Green features are accepted (Language ability, Mother.edu, Stereotyped Speech, Pointing/Gestures, Facial Expression, Social Quality, Unusual Sensory Interest, Negative Behaviors, and Repetitive Stereotyped Behaviors), and red features are rejected (gender, Father.edu, Unusual Eye Contact, Complex Mannerisms, Overactivity, and Anxiety). The yellow features are regarded as edge features (age). Finally, we included 10 accepted variables and edge variables (Language ability, Mother.edu, age, Stereotyped Speech, Pointing/Gestures, Facial Expression, Social Quality, Unusual Sensory Interest, Negative Behaviors, and Repetitive Stereotyped Behaviors) (Table 3).

**TABLE 3:** Comparison of full variables and variables after feature selection.

Type	Quantity	Variable list
Full variable	16	Gender, age, Mother.edu, Father.edu, language ability, stereotyped speech, pointing/gestures, unusual eye contact, facial expression, social quality, unusual sensory interest, complex mannerisms, repetitive stereotyped behaviors, overactivity, negative behaviors, and anxiety
Feature selection	10	Age, Mother.edu, language ability, stereotyped speech, pointing/gestures, facial expression, social quality, unusual sensory interest, negative behaviors, and repetitive stereotyped behaviors

In addition, IDI was calculated to evaluate the contribution of feature processing features (Table 4). The results demonstrated that after feature selection, the prediction performance of SVM, RF, and XGBoost improved to varying degrees (38.20, 4.30, and 10.30%), and the difference was statistically significant (*P* < 0.05). On the other hand, LR model has a certain degree of improvement, but the difference is not statistically significant (*P* > 0.05). Therefore, this study selects 10 variables for the final modeling.

**TABLE 4:** Improvement index before and after feature selection.

Model	IDI	95% CI	*P*-value
LR	0.049	−0.018 to 0.117	0.150
RF	0.043	0.012–0.075	0.006
SVM	0.382	0.244–0.520	<0.001
XGBoost	0.103	0.022–0.184	0.011

### Comparisons between models in internal validation

#### Comparisons of discrimination and calibration

**FIGURE 2:**
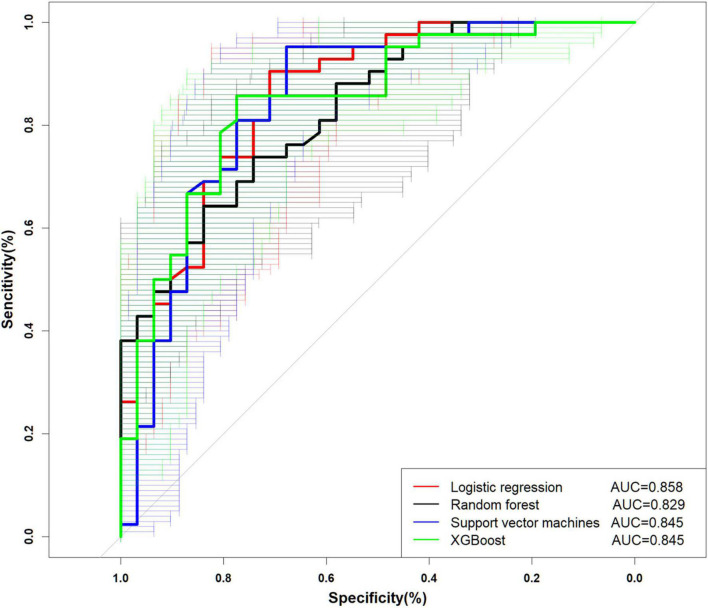
Performance of the diagnostic models.

The performance is displayed in Table 5 and Figure 2. We found that the accuracy of SVM was the best (0.836), and the other three models were more consistent in accuracy; in terms of precision, SVM and XGBoost performed better (0.800, 0.838). LR (0.939) is the best in sensitivity, followed by SVM (0.952). Regarding specificity, SVM, RF, and XGBoost performed more prominently, all higher than LR (0.355). AUC of ML (SVM, 0.835 [95% CI: 0.747–0.944]; RF, 0.829 [95% CI: 0.738–0.920]; XGBoost, 0.845 [95% CI: 0.734–0.937]) vs. traditional LR (0.858 [95% CI: 0.770–0.944]) was not significantly different (Figure 2). Concurrently, no statistical difference was found between the models by De-long test, as revealed in Table 6.

**TABLE 5:** Diagnostic models performance.

Model	Accuracy	Precision	Sensitivity	Specificity	AUC (95% CI)
LR	0.712	0.672	0.976	0.355	0.858 (0.770–0.944)
RF	0.726	0.789	0.714	0.742	0.829 (0.738–0.920)
SVM	0.836	0.800	0.952	0.677	0.845 (0.747–0.944)
XGBoost	0.767	0.838	0.738	0.806	0.845 (0.734–0.937)

**TABLE 6:** De-long test, pairwise comparison between models.

Model	Z	*P-value*
LR vs. RF	1.016	0.309
LR vs. SVM	0.491	0.623
LR vs. XGBoost	0.190	0.848
RF vs. XGBoost	−0.497	0.618
RF vs. XGBoost	−0.244	0.807
SVM vs. XGBoost	0.011	0.990

**FIGURE 3:**
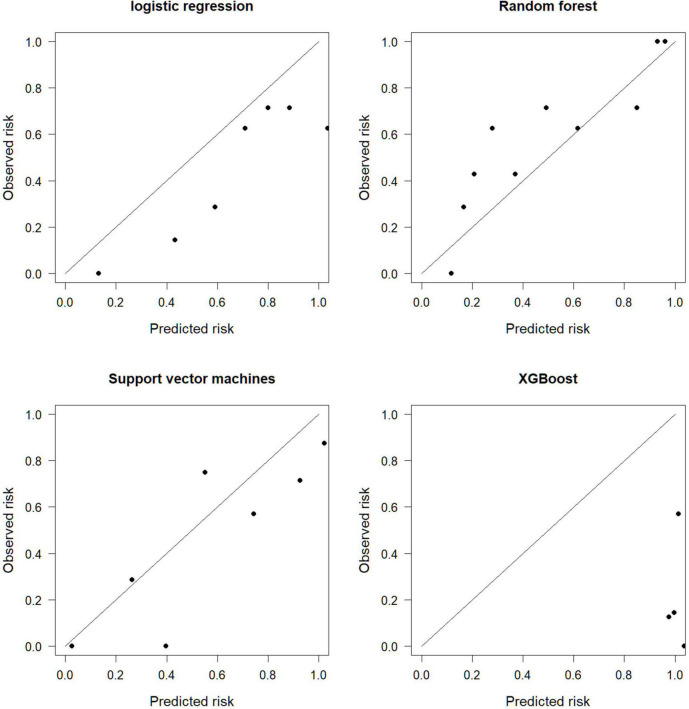
Calibration diagram.

The calibration graph evaluates the consistency between the model prediction results and the actual situation. The SVM is more consistent with the actual situation, and the prediction consistency between LR, RF, and XGBoost is poor (Figure 3).

#### Comparisons of decision curve analysis

**FIGURE 4:**
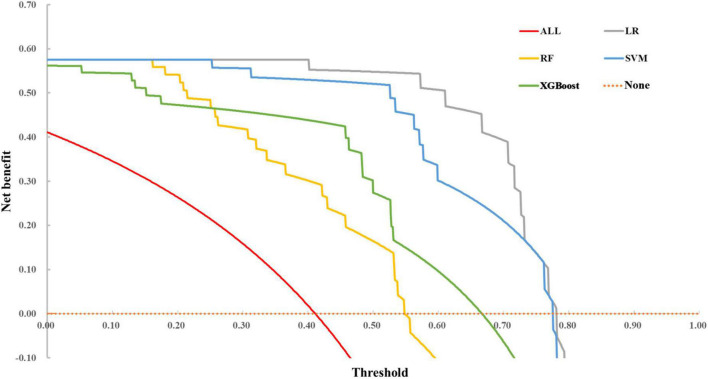
DCA of the four models.

Decision curve analysis curve results are illustrated in Figure 4. The none line on the *X*-axis indicates that all autistic children were non-ID and intervened, with a net benefit of 0. The ALL symbol represents the net benefit at various thresholds, with an assumption that all children with ASD have ID. In most cases, we found that the net benefit of LR was higher than that of SVM, RF, and XGBoost models. However, when the threshold exceeded 0.70, the net benefit of the two ensemble methods returned to zero, while LR and SVM still had an enormous net benefit.

In practice, the threshold is set according to actual requirements. We can use the proportion of non-ID and combined ID in autistic children with different demographic characteristics to calculate and determine the net benefit of different diagnostic models under the set conditions to evaluate the practical value of the model.

To summarize, SVM has higher accuracy, precision, sensitivity, better calibration, and higher benefit over a more extensive threshold range. Therefore, combining discrimination, calibration, and clinical decision curve, we finally choose SVM as the predictive model for whether autistic children are combined with ID.

### Variable importance for diagnostic models

The results for the different importance of each model are demonstrated in Table 7. The SVM model in this study used a non-linear kernel function, and its variable weights were no longer linear, so the SVM importance could not be directly obtained. Therefore, SHAP method was used to evaluate the variable importance of SVM model. For LR, the top five predictors were Mother.edu, Language ability, Repetitive Stereotyped Behaviors, Facial Expression, and Unusual Sensory Interest. It demonstrated that the increase of Mother.edu, Language ability, and Repetitive Stereotyped Behaviors would increase the risk of autistic children being diagnosed with ID. In SVM, the top five predictors are Repetitive Stereotyped Behaviors, Speech, Language ability, Negative Behaviors, and Social Quality. In RF, age, Language ability, Pointing/Gestures, Mother.edu, and Repetitive Stereotyped Behaviors. In XGBoost, the top five predictors were Father.edu, gender, Language ability, Facial Expression, and Unusual Eye Contact. When the importance of predictors in the four models was examined, it was discovered that language ability was vital in each model.

**TABLE 7:** Variable importance order of LR, SVM, RF, and XGBoost.

Variable	Logistic regression		SVM	Random forest		XGBoost		Average rank
	OR	Rank	Rank	IncNodePurity	Rank	Gain	Rank	
Language ability	27.16	2	3	6.07	2	0.35	1	1
Repetitive stereotyped behaviors	4.84	3	1	3.15	5	0.1	3	2
Mother.edu	27.69	1	7	3.18	4	0.06	8	3
Social quality	0.11	7	5	2.46	6	0.09	4	4
Stereotyped speech	0.27	6	2	2.12	8	0.04	9	5
Pointing/gestures	0.07	8	8	3.25	3	0.07	6	5
Negative behaviors	0	10	4	0.41	10	0.13	2	7
Age	5.03	9	10	8.58	1	0.06	7	8
Facial expression	0.64	4	9	1.51	9	0.08	5	8
Unusual sensory interest	0.37	5	6	2.46	7	0.02	10	10

**FIGURE 5:**
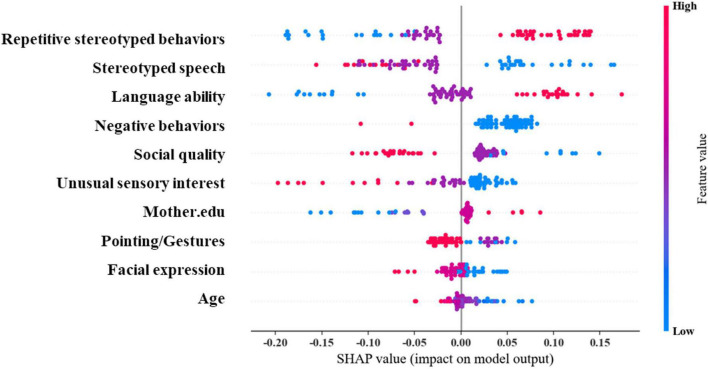
SHAP value and importance of each feature in SVM.

We further used SHAP plots to understand the interpretability of SVM (Figure 5). The results revealed that the top five important predictors are: Repetitive Stereotyped Behaviors, Stereotyped Speech, Language ability, Negative Behaviors, and Social Quality. Among them, the Stereotyped Speech score was more complex in predicting whether autistic children were combined with ID. The outcome is positively correlated with the above predictors within a certain range and negatively correlated beyond this range. The effects of other predictors on long-term outcomes were mainly unidirectional, such as decreasing Repetitive Stereotyped Behaviors score, decreasing Stereotyped Speech score, decreasing Language ability, increasing Negative Behaviors score, Social Quality score, and Unusual Sensory. Furthermore, an increase in the Interest score, a decrease in maternal education, an increase in the Facial Expression score, and an increase in age all increase the likelihood of autistic children being diagnosed with ID. The above results demonstrate that ML method can effectively explain the complex non-linear relationships in the data content.

## Discussion

In this study, based on the socio-demographic information and behavioral observation data of children with ASD, the ML methods were used to construct a diagnostic model for whether autistic children were combined with ID. The results demonstrated that the ML methods could effectively distinguish whether autistic children combined ID. Before building a model requires sufficient data preprocessing, such as outlier identification, missing value filling, normalization, feature selection, etc. The data integrity in this study was all above 99%, indicating that the quality of the data used for diagnosis was good. For variables, this study uses the methods of outlier identification, missing value imputation, normalization, and feature selection to deal with variables. Some studies have pointed out that sufficient data preprocessing should be carried out before training the diagnostic model (49).

Taking the feature selection of this study as an example, AUC-based discrimination in the full-variable prediction model, SVM, RF, and XGBoost all performed lower than the model after feature processing using the Boruta method. This also depicts that feature processing is essential to obtain a more concise and effective classifier. LR, SVM, RF, and XGBoost all show good performance in this study. The AUCs of LR, SVM, RF, and XGBoost are 0.858 (0.770–0.944), 0.829 (0.738–0.920), 0.845 (0.747–0.944), and 0.845 (0.734–0.937), respectively. In the existing evaluation system, there is no evaluation of AUC. The accepted threshold for assessing good classifiers. Rice et al. proposed that AUC can be converted to effect sizes, such as Cohen’s *d* and Pearson’s r*_*pb*_* (50).

In this study, the Cohen’s *d* values of LR, SVM, RF, and XGBoost were 1.515, 1.344, 1.436, 1.436, and r*_*pb*_* were 0.604, 0.558, 0.583, and 0.583, respectively. According to Cohen’s *d* intensity criteria, our diagnostic models are equivalent to high impact levels. According to the standard of impact strength on r*_*pb*_*, traditional LR demonstrated high correlation, and SVM, RF, and XGBoost revealed moderate correlation levels. In addition, some studies also show that the performance of ML and regression models are comparable (27, 51, 52). However, the difference between ML and traditional regression models in this study is not obvious, which is also different from most current studies. Possible explanations are as follows: ML is good at processing big data, so complex rules may not be found in the case of limited data. At the same time, research on autism-related diagnostic models focuses on high-cost imaging materials such as MRI, while this study focuses on relatively easily available behavioral observations and socio-demographic data (37, 53, 54). In addition, selecting the best variable combination also has certain difficulties. Pepe et al. suggest that even with the same study category and study data, the variable combinations of influencing factors and diagnostic models may be extremely contradictory (55). In other words, a factor may be closely related to the disease, but its contribution to the diagnostic model may be minor. As a result, we can conclude that the diagnostic model obtained in this study is not optimal but only performs well in this sample. At the same time, interpreting the final influencing factors still requires relevant clinical knowledge and experience to solve and explain the problem.

Our research found that among the best SVM models, Repetitive Stereotyped Behaviors, Stereotyped Speech, and Language ability were the top three key variables. Repetitive Stereotyped Behaviors and Stereotyped Speech belong to RRBs (Restrictive and Repetitive Behaviors). Although RRB is one of the two core symptoms of children with ASD, it is not unique to autism. Other neurodevelopmental disorders and even ordinary children may also have RRB manifestations (56). Therefore, the diagnostic model constructed in this study is only suitable for children diagnosed with autism. For a long time, the relationship between RRBs and IQ has not reached a consensus (57, 58). The diagnostic model constructed by ML in this study suggests that the higher the score of repetitive, stereotyped behavior in children with autism, the greater the possibility of combined ID. However, the precise reason is unknown due to its complex biological and psychological mechanism (59). In addition to impairing intelligence, RRBs can impair the physical and mental health of individuals with autism (59), affect social and daily living skills in children (60) and adolescents (61), and predict the onset of anxiety (62, 63). Fortunately, the severity of repetitive stereotypes can be alleviated through intervention training (64), but whether RRB intervention can improve IQ levels requires further research.

Although language disorder (LD) is no longer a core symptom of ASD, it is one of the common comorbidities of ASD (1). About 63% of autistic children have LD (65). Interestingly, in all four models of this study, Language ability demonstrated significant significance for the diagnosis of ASD comorbid ID. In other words, language ability is an important diagnostic factor for whether children with ASD have combined ID, and the worse the language ability, the greater the possibility of combined ID. In a broad sense, language ability includes language perception and expression ability (66). Language ability in this model refers to the latter, while the Verbal Comprehension Index (VCI) is directly included in WISC-IV, and the score of language comprehension will become calculate the part of total IQ (67). Many studies have revealed that non-verbal IQ may be a strong positive predictor of language ability in children with ASD (68, 69). This study suggests that language ability also positively impacts IQ, so strengthening language intervention for children with ASD may also have a positive effect.

The average level of the mother’s educational level ranks third in the four models. That is to say, whether autistic children are combined with ID may have a certain relationship with the mother’s educational level. The higher the mother’s education level, the lower the likelihood of autistic children with ID. The lower education level of parents may affect their awareness of ASD, thereby delaying the diagnosis of children with ASD (70), and mothers with lower education levels have greater parenting pressure and are more likely to develop anxiety and depression (71). Moreover, mothers with higher education levels can better regulate their emotions, actively carry out rehabilitation training for their children, and improve their children’s abilities (72, 73). This conclusion is not a judgment of causality, and the specific mechanism remains to be further studied, which is different from the overturned “refrigerator mother” theory (74).

Assessing the clinical utility of diagnostic models can guide clinical practice. DCA was performed in this study, combining discrimination, calibration, and DCA, and the results showed that SVM was relatively superior in terms of clinical benefit and ability to discriminate against autistic children with ID. Medical and health workers can collect socio-demographic data and behavioral observations of autistic children and use the recommended SVM model for children with autism in grassroots units that cannot conduct systematic intelligence assessments (such as the WISC-IV test, etc.). Preliminary diagnosis of ID is the premise of a stepped care and personalized health approach for children with different types of ASD (75).

Our study may have some potential advantages. First, we used demographic and behavioral observational data from a population cross-section to construct a diagnostic model with low costs of model construction and easy access to diagnostic data. Therefore, initial diagnostic screening may be applicable to primary care physicians. Second, the optimal SVM model is computationally efficient and can be quickly computed in real time during practical application. In addition, we conducted a relatively complete data pre-processing work to ensure the processing effect of the prediction model and provide a solid foundation for the model architecture. Finally, we conducted a comprehensive evaluation of the prediction model, including traditional metrics (identification and calibration), and clinical utility analysis (decision curves and prediction curves). The SHAP method was also used with a view to discussing in further depth the interpretability of the ML model. Also we followed the standard reporting procedures for prediction models described in TRIPOD (76).

The limitation of this study is that the sample size is relatively small. If the sample size continues to increase, the model trained by ML will be more convincing. Concurrently, this study only evaluates the generalization ability of the diagnostic model through internal verification. Further research must be conducted in other perform external validation on ASD population in the region or subsequent children on the ASD in the region. The diagnostic model is based on the current behavioral observation to evaluate the current IQ, which solves the current situation that the intelligence test cannot be fully promoted in real situations. However, children with ASD are frequently diagnosed in early childhood, and intelligence evaluation results at this stage are not stable. Therefore, predicting school-age IQ through behavioral observations of children with ASD in early childhood is more in line with clinical reality, which needs to establish a follow-up cohort of autistic children.

## Conclusion

Based on the data of children with ASD in Zhejiang, China, the ML model can effectively distinguish whether autistic children are combined with ID. Given the degree of discrimination, calibration, and clinical usefulness, we believe SVM is the best model for screening autistic children with ID.

## Data availability statement

The raw data supporting the conclusions of this article will be made available by the authors, without undue reservation.

## Author contributions

CS conceived the study and wrote the draft of the manuscript. Z-QJ conducted literature searches and completed the data analysis. L-FH and X-LL engaged in behavioral observation. W-HL, Y-YW, and W-YJ conducted the IQ test. Z-WZ critically revised the manuscript. All authors contributed to the article and approved the submitted version.

## Appendix

**Table:** Schedule 1 Assignment table.

Variable	Category	Assignment
Gender	Categorical	Male = 1; Female = 0
Language ability	Categorical	Pre-verbal/single words = 1; Phrase speech = 2; Fluent speech = 3.
Father’s education attainment	Categorical	Primary school = 1; Secondary school = 2; High school = 3; College/university = 4; Graduate and above = 5.
Mother’s education attainment	Categorical	Primary school = 1; Secondary school = 2; High school = 3; College/university = 4; Graduate and above = 5.
Age	Continuous	–
Stereotyped speech	Continuous	–
Pointing/gestures	Continuous	–
Unusual eye contact	Continuous	–
Facial expression	Continuous	–
Social quality	Continuous	–
Unusual sensory interest	Continuous	–
Complex mannerisms	Continuous	–
Repetitive stereotyped behaviors	Continuous	–
Overactivity	Continuous	–
Negative behaviors	Continuous	–
Anxiety	Continuous	–
Outcome	Categorical	Non-ID = 1; ID = 0
